# No biomedical engineering policy means no sustainability in health systems: perspective through a case of local biomedical training program in Tanzania

**DOI:** 10.11604/pamj.2024.49.40.43221

**Published:** 2024-10-14

**Authors:** Suniva Sivero Celestin Haule, Valentino Mvanga, Swabaha Aidarus Yusuph, Hayoung Kim, Hansol Park, Kyoung Kyun Oh

**Affiliations:** 1 Directorate of Diagnostic Services, Ministry of Health, Dodoma, Tanzania,; 2 Korea Foundation for International Healthcare, Tanzania Office, Dar es Salaam, Tanzania

**Keywords:** Health system strengthening, biomedical engineering, in-service training, on-the-job training, official development assistance, Korea Foundation for International Healthcare, Tanzania

## Abstract

While the significance of strengthening the biomedical workforce in resource-limited settings has been widely acknowledged, there remains a paucity of information specific to the local context. In this regard, we underscore the importance of formulating a biomedical engineering policy based on empirical evidence. To provide such evidence, we conducted an analysis of the government-led biomedical training program in Tanzania, titled 'Capacity Enhancement of Medical Equipment Technical Services (CEOMETS)'. The program demonstrated statistically significant outcomes at the individual level, as evidenced by a T-test comparing pre- and post-self-assessments of self-efficacy indicators from 2018 to 2022 (n=121) (***p < 0.001). However, the program's impact was largely confined to individual-level capacity building, with limited effects at the systemic level. The findings also revealed substantial structural and institutional gaps, as well as a lack of engagement from global stakeholders and key actors in the field of biomedical engineering. From this perspective, therefore, we emphasize the necessity of two key preconditions. First, on a national level, there is an urgent need to develop strategic plans, guidelines, and workforce policies that formally integrate biomedical engineers into efforts to strengthen the health system in Tanzania. The other arm is a way to call for action to the global health players to engage more actively in supporting biomedical engineering development in such contexts. Ultimately, this perspective paper aims to set a milestone in establishing the policy-making process regarding biomedical engineering as a health policy issue in Tanzania.

## Perspectives

Biomedical engineering (BME) plays a vital role in managing medical equipment, facilitating timely diagnosis, proper treatment, and improved prognosis [1]. Biomedical engineering is regarded as a convergence discipline and a crucial tool for addressing some of the world´s most challenging health problems through an 'intersectional' approach [2-4]. Strengthening BME can pave the way toward achieving universal health coverage (UHC) and promoting more equitable healthcare. Specifically, BME has the potential to maximize the impact of UHC, especially in low- and middle-income countries (LMICs), in relation to health system strengthening (HSS) [4].

However, BME in resource-limited settings faces challenges similar to other global health issues. Many LMICs are disproportionately reliant on imported medical devices, with over 95% of medical devices in public hospitals being imported [5]. Poor medical equipment maintenance is largely due to a lack of well-trained biomedical engineers, inconsistent sources of donated equipment, and donations of medical equipment without a prior understanding of the local context [6]. Thus, focusing on BME, particularly in human resource capacity-building, is a cost-effective approach to strengthening health systems in less resilient settings. Biomedical engineering (BME) can serve as a supplementary tool for holistic HSS, ensuring the availability and quality of essential health services.

There have been notable internal efforts to address the challenges mentioned above. In 2015, a local BME workforce training program called the 'Capacity Enhancement of Medical Equipment Technical Services (CEOMETS)´ was launched in Tanzania. This program has been implemented annually to train local BME workforce. In September 2022, Tanzanian biomedical engineers and technicians established the 'Association of Medical Engineers and Technicians in Tanzania (AMETT)' to promote institutional strengthening, individual capacity-building, and systematic sustainability. The Ministry of Health (MOH) of the Government of Tanzania (GoT) has announced plans to employ more BME workers, as the country currently has only 350 biomedical engineers as of 2023. According to the latest public announcement, the GoT aims to fill the gap of over 9,000 biomedical engineers to cope with the growing demand for medical equipment in hospitals nationwide [7].

While there have been significant improvements over the past decade, it is evident that Tanzania still has a long way to go. Despite the GoT´s ambitious goals, there remains a lack of studies on local BME. Additionally, there has been little assessment of local BME training programs, which leaves a gap in the literature. To address this gap, we aimed to analyze the direct outcomes of the CEOMETS training program. By presenting and discussing its results, we hope to identify current challenges and opportunities for BME from a systematic perspective in Tanzania. Ultimately, this study aims to contribute to the policy-making process regarding biomedical engineering as a global health policy issue in Tanzania.

**Figure 1:**
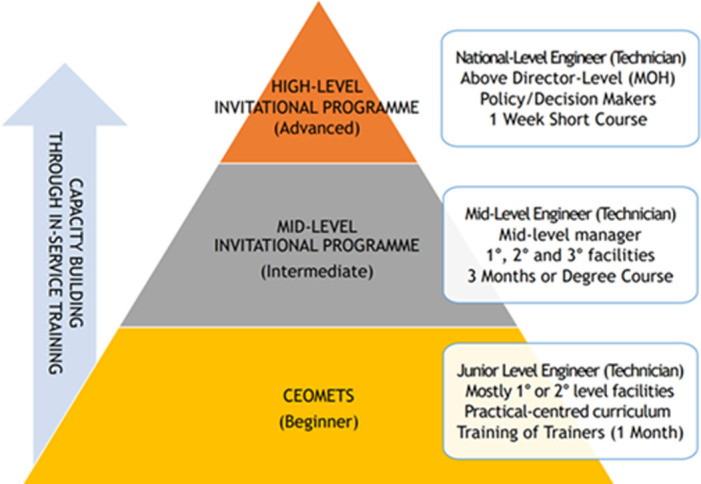
conceptual diagram of an invitational program for the biomedical workforce

**Capacity Enhancement of Medical Equipment Technical Services training program overview:** the government-led CEOMETS program is part of the invitational training initiative known as the 'Dr Lee Jong-wook Fellowship Program,' supported by the Korea Foundation for International Healthcare (KOFIH) under the Ministry of Health and Welfare of the Government of Korea (Figure 1). Capacity Enhancement of Medical Equipment Technical Services aims to enhance the capacity for medical equipment maintenance and repair services among the junior-level biomedical workforce.

The CEOMETS training program consists of a four-week intensive course with a total of 178 credit hours. Of these, approximately 76 credit hours (42.7%) are dedicated to theoretical lectures, 82 credit hours (46.1%) to practical sessions, including 16 hours of maintenance field trips to hospitals, and 20 credit hours (11.2%) to evaluation, discussion, and presentations. The lectures cover essential principles of medical equipment commonly used in health facilities. Following the lectures, trainees have the opportunity to repair broken equipment using a training-of-trainers model (Annex 1), with senior-level engineers from the public sector serving as trainers. This approach is particularly beneficial for personnel addressing maintenance-related challenges.

On the final day of the course, trainees are required to develop a maintenance plan for their home facilities, with a focus on sustainability. As illustrated in Figure 1, one or two high-performing trainees from the local CEOMETS training may be selected to attend a three-month invitational BME course in the Republic of Korea. There is also an opportunity for mid-level managers to pursue a master´s degree through this program. In addition, high-level officials or directors from the Ministry of Health may participate in a one-week high-level invitational program that focuses on leadership, decision-making, and governance skills, aiming for a broader impact on BME.

**Figure 2:**
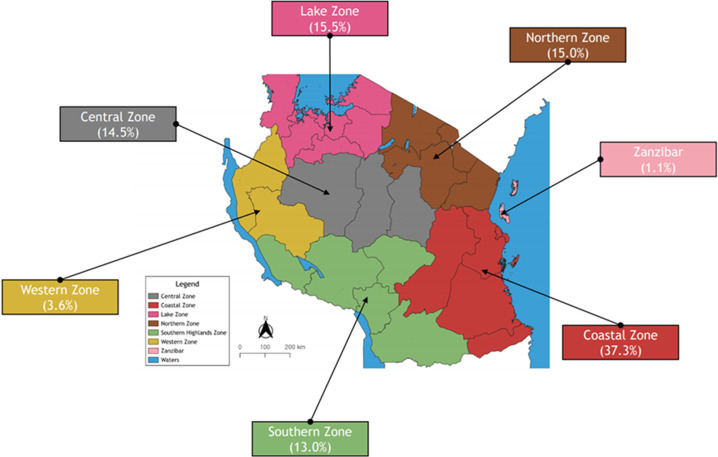
geographical distribution of CEOMETS trainees

**Analytic results of CEOMETS:** between 2015 and 2022, a total of 193 trainees participated in the CEOMETS program across the United Republic of Tanzania (sex ratio: 70% male, 30% female). The geographical distribution of trainees includes the Coastal Zone (37.3%), Lake Zone (15.5%), Northern Zone (15.0%), Central Zone (14.5%), Southern Zone (13.0%), Western Zone (3.6%), and Zanzibar (1.1%) (Figure 2). Of the participants, 18.1% (35 individuals) held bachelor's degrees (engineers), while 81.9% (158 individuals) had diplomas (technicians). As of 2023, approximately 75.1% of the trainees remain active within the BME ecosystem.

Although the training is conducted in the capital city, Dodoma, participants are drawn from various regions, ranging from the Coastal Zone to the Western Zone and even the Zanzibar Archipelago. The high representation from the Coastal Zone is likely due to its population density and urbanization. In contrast, participation from the Western Zone, including the southwest and southern highlands, has been relatively low. This disparity may be attributed to a shortage of qualified candidates and challenges in accessing the training facility.

**Figure 3:**
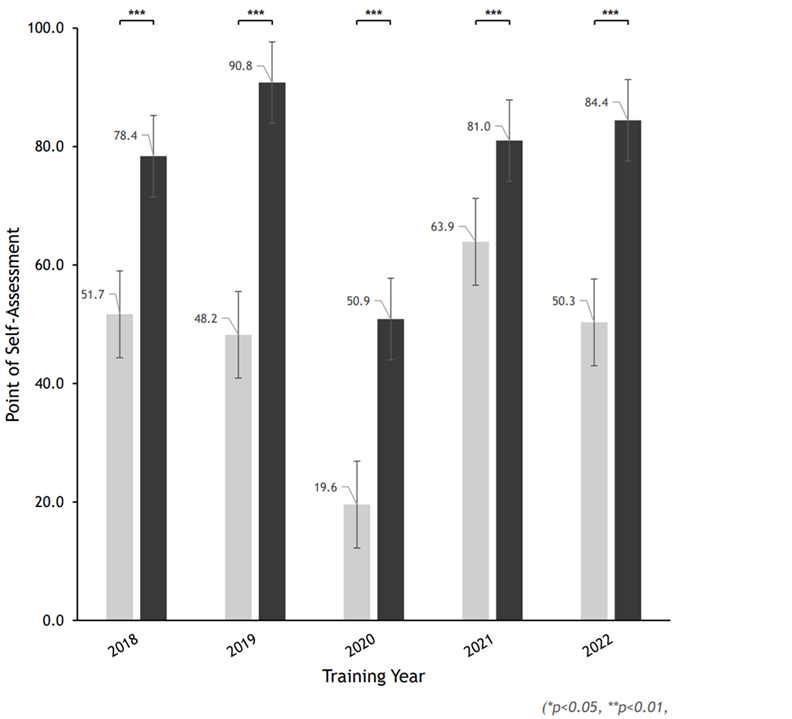
the pre- and post-self-assessment results of CEOMETS (2018-2022)

We analyzed self-efficacy indicators statistically using pre- and post-assessment data collected between 2018 and 2022 (n=121). The data were gathered as part of the program´s performance evaluation. A paired T-test was employed to assess the training's effectiveness, with significance set at p < 0.05. The results revealed a highly significant improvement in post-assessment scores compared to pre-assessment scores across the five-year period (***p < 0.001) (Figure 3). Specifically, annual performance showed notable improvement: 2018 (n=25, t=-8.51), 2019 (n=25, t=-21.40), 2020 (n=25, t=-9.42), 2021 (n=29, t=-12.53), and 2022 (n=17, t=-9.64), all significant at ***p < 0.001 (Table 1). On average, pre-assessment scores were around 60, while post-assessment scores consistently exceeded 80, reflecting an approximate 20-point improvement. The largest discrepancy between pre- and post-scores occurred in 2019.

**Table 1:** a paired sample T-test results of pre- and post-self-assessment (2018-2022)

Training year	Pre/post	n	Mean	Differences	SD	t	p-value
2018	Pre	25	51.7	26.7	12.4	-8.51	***< 0.001
	Post	25	78.4		9.0		
2019	Pre	25	48.2	42.6	7.5	-21.40	***< 0.001
	Post	25	90.8		6.3		
2020	Pre	25	19.6	31.3	7.0	-9.42	***< 0.001
	Post	25	50.9		14.7		
2021	Pre	29	63.9	24.2	8.5	-12.53	***< 0.001
	Post	29	88.1		5.7		
2022	Pre	17	50.3	34.1	11.5	-9.64	***< 0.001
	Post	17	84.4		8.3		

† SD= Standard deviation

**Implications and recommendations for CEOMETS:** the leading sound outcome was fostering talented BME personnel and improving technicality among entry-level junior trainees with highly relevant training content. Indeed, the statistical result of the self-assessment indicator addresses that the program was effective at individual-level capacity building. Given that chances for those who studied in the BME at educational institutions are not enough to deal with case studies, on-the-job training (OJT) or in-service training after graduation, this activity played a pivotal role in enhancing individual capabilities through practical and technical experiences. Furthermore, this program is the only available in-service training offered in Tanzania. Consequently, the program motivates participants to perform well during the training as trainees with excellent learning achievements may progress to the next level of invitational programs conducted abroad.

We recognized many challenges and limitations throughout operating and evaluating the training program and the situation of national-level BME policies. As aforementioned, there is a limitation in training biomedical engineers and technicians nationwide, given only one month opportunity. Although the participants are from different geographical areas, there is room for improvement to incorporate people from other mountainous, remote regions. Additionally, there is no in-service training for those who have already participated in the program in previous years. In-service training is vital as it improves productivity and fosters better work amongst the trainees. There is also a limitation in the training content and the quality of content as there are only a few trainers who can give lectures and practical training on medical equipment. Having different experts not only nationally but also from neighboring countries may help share knowledge and experience for the quality of training. Last but not least, adequate funds need to be appropriately allocated and well-managed by the government for better outcomes and sustainability of the training program.

There are also limitations in the pre-and post-tests, which are mainly self-assessed yearly by the participants. The content of the courses that are taught differs slightly by year, which may affect the overall satisfaction of the trainees. There is a need for an additional assessment to understand and evaluate the impact of such local training to better understand what needs to be improved for future training. Trainers need to take the initiative to follow up on past trainees to understand what has been successful and what has been beneficial to the local trainees. In addition, there is a need to assess the quality of training and how one could apply the contents learned from the training to take real-life action once they return to their facility. Action plans of what they plan to do once they return to their facility and how they will share their knowledge from the training with their colleagues could be an additional assignment for the participants.

Apart from the limitations of CEOMETS, there are underlying fundamental limitations in the BME field in Tanzania. The vitality of biomedical technology and engineering, which provide the entire population with quality essential health services, is often underestimated. This phenomenon has resulted in having over 95% of the medical equipment in public hospitals imported, with no local production [1,8]. A myriad of medical equipment is not properly functioning due to an insufficient supply chain of spare parts, an inability to fix and manage the equipment, constraints on the budget, and a weak industrial foundation. Leading hurdles lie in the high initial and running costs and the fact that many of the latest medical devices are not designed to operate under limited conditions such as unstable power supply, humidity, dust, et cetera [1].

Technology in Tanzania mainly involves manufacturing low-tech and disposable items such as ultrasound gel or mosquito nets. Although state-of-the-art gadgets are known to be often undesired, and mainstream items are of greater use, in reality, due to scarce choices, only high-tech devices with high prices but low quality are available today. Donated apparatus, sometimes unutilized due to lack of demand or outdated, is an enormous burden to many LMICs. The country highly depends on donated medical equipment, but often, there is no efficient funding for operation, maintenance, and training. Due to the lack of proper needs assessment before donation and provision, inadequate devices are often provided with no appropriate guidelines and easily interpretable manuals [9]. At times, the equipment and manuals are written in a foreign language without local translation, which can be a bottleneck in not only fixing but also in use for local health workers. Hence, there is a need for a needs-based and culturally appropriate way of supporting LMICs, especially regarding medical equipment donation, as it may cause an adverse situation regardless of the benevolent intention. Although the topic of ‘technology and knowledge transfer´ to LMICs has been viral over the past decades, a national-level management system is needed for effective coordination and regulation [10]. These include open-sourcing technology to work around copyright laws and subsidizing devices [1,5,9,11]. Such national-level management systems must go hand in hand with ´technology and knowledge transfer´ activities from partner organizations and countries for sustainable technological advancement. This will also contribute to strengthening the health system, as medical equipment plays a significant role from diagnosis to treatment of the patients.

Endogenous issues: institutional and structural limitations: the BME education with knowledge of science and technology plays a critical role in designing, maintaining, and repairing medical devices [10,12]. Universities nurture biomedical engineers by granting bachelor´s degrees, and technical schools (colleges) foster technicians by granting diplomas. Five institutions in Tanzania offer the BME education. However, only two (Muhimbili University of Health and Allied Sciences (MUHAS) and Arusha Technical College (ATC)) grant bachelor´s degrees (Annex 2). In other words, the lack of BME teaching institutions has resulted in nurturing a few cohorts of the BME workforce who have to increase their technical and political voices. Also, infrastructure, workshop spaces for practices, and connections with sound career paths after graduation are obscure. These factors lead to the competency of BME personnel and result in low recognition, poor condition, and treatment of BME personnel at health facilities in general. Biomedical engineering credential, certification, or license systems have not yet been planted. Although some have certificates in neighboring fields, such as electrics, the BME workforce does not have national licensing or appropriate certificate management systems. The absolute number of BME engineers and technicians is very scarce. There are allegedly 158 technicians only active, 35 estimated bachelor’s level in public areas, and only four master´s level qualifications as of 2022. No one has been granted a PhD level education yet. Unfortunately, as of today, there are only two master´s level qualified tutors at MUHAS. Clearly, these values show that there have been and still have institutional and structural limitations to cultivate the next generation of biomedical engineers. It is necessary to raise awareness and boost visibility for the nation to invest in BME, as it will have an enormous and immediate impact on its health system.

**Exogeneous issues: policy-making and global partnership:** policy-making actions and partnerships are fundamental agendas for achieving Sustainable Development Goals (SDGs). Sustainable development goals 17, in particular, is focused on global partnerships where technology and engineering play a significant role [10,12,13]. However, national policy, strategic plans and guidelines, and workforce plans for the BME that reflect biomedical engineers are currently in need, and the lack of such frameworks accounts for truncated and fragmented global cooperation and partnerships. Without such a basis, global partners and their contribution to the BME would inevitably not be an evidence-based action and ultimately would result in less sustainable collaboration under ex-potential pressure. Of course, efforts have existed to fill the administrative gaps, but there is still a large room for improvement. Additionally, a limited number of players are interested in BME, which is the leading challenge. Only a few players, such as the Republic of Korea, Switzerland, the Netherlands, and the United States, are the de facto actors in the BME field in Tanzania as of 2022. All in all, there is an urgent need for governmental action that can boost the visibility of biomedical engineers, as well as sustainability in terms of training and maintaining the BME workforce to strengthen the health system of Tanzania.

## Conclusion

Our analysis demonstrates CEOMETS´ effectiveness in the capacity-building of the junior BME workforce in Tanzania, particularly through significant improvements in pre- and post-training scores over five years. The program has facilitated nationwide knowledge dissemination, drawing participants from various regions. However, despite these benefits, challenges remain, including the need for national-level policies to address curriculum gaps (in reflection of comparative study amongst three countries: Republic of Korea (YONSEI University), United Kingdom (King’s College London), and United Republic of Tanzania (MUHAS) [14-16] (Annex 3), workforce management, and qualifications. Moreover, greater integration with health system building blocks and collaboration with donor partners are essential for sustainable improvements. Establishing quality control measures, such as national proficiency exams and professional standards, is crucial to ensure competency. While CEOMETS has made notable strides, it sheds light on the fact that comprehensive reforms in policy, education, and workforce development are necessary for long-term impact.

## Annexes

**Annex 1**: practical training session through training of trainer’s method (PDF-206KB)

**Annex 2**: the number of institutions offering biomedical engineering education and the number of yearly cohorts of the biomedical engineering education workforce in Tanzania (PDF-116KB)

**Annex 3**: comparison table of the biomedical engineering courses for three different countries (Korea, UK, Tanzania) (PDF-178KB)

## References

[1] Wall K (2010). Engineering: Issues, challenges and opportunities for development. UNESCO report.

[2] Douglas TS (2012). Biomedical engineering education for developing countries. IEEE Technol Soc Mag.

[3] Douglas TS (2011). Biomedical engineering education in developing countries: research synthesis. Annu Int Conf IEEE Eng Med Biol Soc.

[4] LeDuc P, Agaba M, Cheng CM, Gracio J, Guzman A, Middelberg A (2014). Beyond disease, how biomedical engineering can improve global health. Sci Transl Med.

[5] Lustick DR, Zaman MH (2011). Biomedical engineering education and practice challenges and opportunities in improving health in developing countries 2011 Atlanta Conference on Science and Innovation Policy. IEEE.

[6] Ayah R, Ong´ech J, Mbugua EM, Kosgei RC, Waller K, Gathara D (2020). Responding to maternal, neonatal and child health equipment needs in Kenya: a model for an innovation ecosystem leveraging on collaborations and partnerships. BMJ Innov.

[7] Daily News (2023). Govt to employ more biomedical engineers.

[8] Malkin RA (2007). Design of health care technologies for the developing world. Annu Rev Biomed Eng.

[9] McDonald S, Fabbri A, Parker L, Williams J, Bero L (2019). Medical donations are not always free: an assessment of compliance of medicine and medical device donations with World Health Organization guidelines (2009-2017). Int Health.

[10] Clifford KL, Zaman MH (2016). Engineering, global health, and inclusive innovation: focus on partnership, system strengthening, and local impact for SDGs. Glob Health Action.

[11] Richards-Kortum R (2009). Biomedical Engineering for Global Health.

[12] De Maria C, Díaz Lantada A, Jämsä T, Pecchia L, Ahluwalia A (2022). Biomedical engineering in low-and middle-income settings: analysis of current state, challenges and best practices. Health Technol (Berl).

[13] United Nations; Department of Economic and Social Affairs; Sustainable Development Goals (2023). Strengthen the means of implementation and revitalize the Global Partnership for Sustainable Development.

[14] (2023). Yonsei University; Department of Biomedical Engineering. Curriculum.

[15] Kings College London (2023). Undergraduate Study, Biomedical Engineering.

[16] Muhimbili University of Health and Allied Sciences (2019). Competency Based Curriculum for Bachelor of Biomedical Engineering. Dept Physiotherapy, School of Medicine, MUHAS.

